# Supplementary material for: Calcitriol, an Active Form of Vitamin D3, Mitigates Skin Barrier Dysfunction in Atopic Dermatitis NC/Nga Mice
Source: Int J Mol Sci. 2023 May 27;24(11):9347. doi: 10.3390/ijms24119347 (PMC10253123; doi:10.3390/ijms24119347)
Supplement: Supplementary file 1 [file ijms-24-09347-s001.zip › ijms-2381178-supplementary.pdf]

Supplementary Data

Calcitriol, an Active Form of Vitamin D3, Mitigates Skin Barrier Dysfunction in Atopic Dermatitis NC/Nga Mice

Yoshie Umehara [1], Juan Valentin Trujillo-Paez [1], Hainan Yue [1,2], Ge Peng [1,2], Hai Le Thanh Nguyen [1,2], Ko Okumura [1], Hideoki Ogawa [1] and François Niyonsaba [1,3,*]

[1] Atopy (Allergy) Research Center, Juntendo University Graduate School of Medicine, Tokyo 113-8421, Japan; y-umeha@juntendo.ac.jp (Y.U.); t-valentin@juntendo.ac.jp (J.V.T.-P.); h-yue@juntendo.ac.jp (H.Y.); g-peng@juntendo.ac.jp (G.P.); ha-nguyen@juntendo.ac.jp (H.L.T.N.); kokumura@juntendo.ac.jp (K.O.); ogawa@juntendo.ac.jp (H.O.)

[2] Department of Dermatology and Allergology, Juntendo University Graduate School of Medicine, Tokyo 113-8421, Japan

[3] Faculty of International Liberal Arts Global Health Studies, Juntendo University, Tokyo 113-8421, Japan

* Correspondence: francois@juntendo.ac.jp; Tel.: +81-3-5802-1591; Fax: +81-3-3813-5512

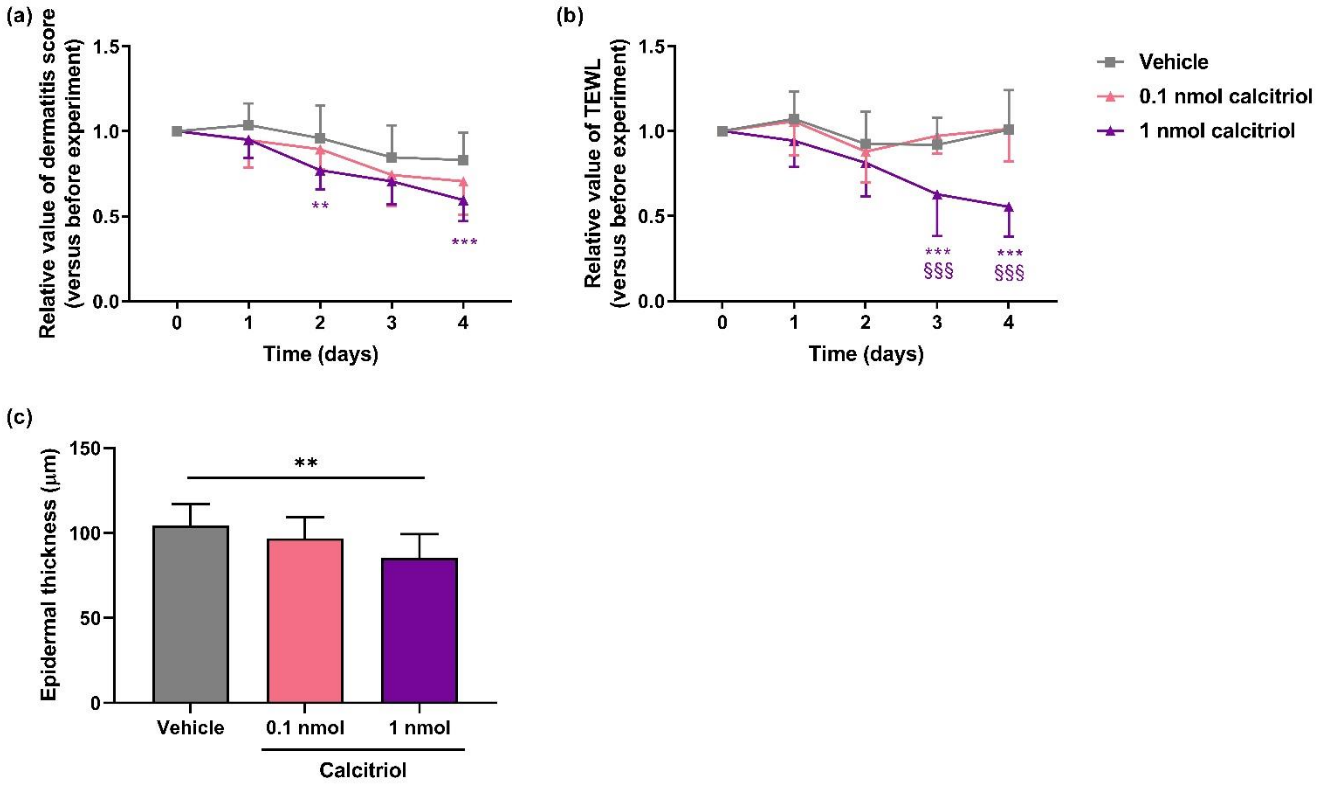

Supplementary Figure S1. The therapeutic role of calcitriol in NC/Nga mice with atopic dermatitis.

(a) and (b) Dfb-induced atopic dermatitis model in NC/Nga mice were applied topically with 0.1 nmol calcitriol, 1 nmol calcitriol or vehicle, and dermatitis score (a) and TEWL (b) were measured once daily for 4 days. The data represent the means ± SDs in each group (n = 7–9). $^{**}P<0.01$ and $^{***}P<0.001$ compared with between vehicle and 1 nmol calcitriol groups; $^{§§§}P<0.001$ compared between 0.1 nmol and 1 nmol calcitriol groups by two-way ANOVA with Tukey's multiple comparisons test. (c) Epidermal thickness on day 4 of lesional skin of NC/Nga mice with atopic dermatitis treated with 0.1 nmol calcitriol, 1 nmol calcitriol or vehicle. The data represent the means ± SDs in each group (n = 9). $^{**}P<0.01$ compared with between vehicle and 1 nmol calcitriol groups by one-way ANOVA with Tukey's multiple comparisons test.
